# Genetic Diversity and Low Therapeutic Impact of Variant-Specific Markers in HIV-1 Pol Proteins

**DOI:** 10.3389/fmicb.2022.866705

**Published:** 2022-07-14

**Authors:** Paloma Troyano-Hernáez, Roberto Reinosa, Africa Holguín

**Affiliations:** HIV-1 Molecular Epidemiology Laboratory, Department of Microbiology, Instituto Ramón y Cajal de Investigación Sanitaria (IRYCIS), Hospital Universitario Ramón y Cajal, CIBER en Epidemiología y Salud Pública (CIBERESP), Red en Investigación Translacional en Infecciones Pediátricas (RITIP), Madrid, Spain

**Keywords:** HIV-1, Pol, protease, integrase, conservation, variants, resistance, reverse transcriptase

## Abstract

The emergence and spread of new HIV-1 variants pose a challenge for the effectiveness of antiretrovirals (ARV) targeting Pol proteins. During viral evolution, non-synonymous mutations have fixed along the viral genome, leading to amino acid (aa) changes that can be variant-specific (V-markers). Those V-markers fixed in positions associated with drug resistance mutations (DRM), or R-markers, can impact drug susceptibility and resistance pathways. All available HIV-1 Pol sequences from ARV-naïve subjects were downloaded from the United States Los Alamos HIV Sequence Database, selecting 59,733 protease (PR), 6,437 retrotranscriptase (RT), and 6,059 integrase (IN) complete sequences ascribed to the four HIV-1 groups and group M subtypes and circulating recombinant forms (CRFs). Using a bioinformatics tool developed in our laboratory (EpiMolBio), we inferred the consensus sequences for each Pol protein and HIV-1 variant to analyze the aa conservation in Pol. We analyzed the Wu–Kabat protein variability coefficient (WK) in PR, RT, and IN group M to study the susceptibility of each site to evolutionary replacements. We identified as V-markers the variant-specific aa changes present in >75% of the sequences in variants with >5 available sequences, considering R-markers those V-markers that corresponded to DRM according to the IAS-USA2019 and Stanford-Database 9.0. The mean aa conservation of HIV-1 and group M consensus was 82.60%/93.11% in PR, 88.81%/94.07% in RT, and 90.98%/96.02% in IN. The median group M WK was 10 in PR, 4 in RT, and 5 in IN. The residues involved in binding or catalytic sites showed a variability <0.5%. We identified 106 V-markers: 31 in PR, 28 in RT, and 47 in IN, present in 11, 12, and 13 variants, respectively. Among them, eight (7.5%) were R-markers, present in five variants, being minor DRM with little potential effect on ARV susceptibility. We present a thorough analysis of Pol variability among all HIV-1 variants circulating to date. The relatively high aa conservation observed in Pol proteins across HIV-1 variants highlights their critical role in the viral cycle. However, further studies are needed to understand the V-markers’ impact on the Pol proteins structure, viral cycle, or treatment strategies, and periodic variability surveillance studies are also required to understand PR, RT, and IN evolution.

## Introduction

HIV is one of the most genetically diverse pathogens due to its high recombination and mutation rates and its rapid replication rate (Hemelaar, 2012; Hemelaar et al., 2019). HIV mutations during replication are favored by the error-prone polymerization by the HIV reverse transcriptase (RT) that lacks proofreading exonuclease activity (Roberts et al., 1988; Bebenek et al., 1993). HIV-1 is responsible for most HIV infections worldwide. It is divided into four groups according to genetic homology: M (major or main), N (non-M, non-O) (Simon et al., 1998), O (outlier) (De Leys et al., 1990), and P (Plantier et al., 2009). Group M is the main HIV group related to the current HIV global pandemic (Hemelaar et al., 2019). This group is subdivided into 10 subtypes (A–D, F–H, and J–L) and 8 sub-subtypes (A1, A2, A3, A4, A5, A6, F1, and F2) (Robertson et al., 2000; Salminen et al., 2000; Yamaguchi et al., 2020), at least 118 circulating recombinant forms (CRFs) (Los Alamos National Laboratory, 2021), and countless unique recombinant forms (URF). The Pol protein has been associated with differences in the replication capacity and disease progression of the different subtypes (Nagata et al., 2017).

The HIV-1 *pol* gene encodes the three enzymes needed for viral replication: protease (PR), RT, and integrase (IN). These proteins have essential roles in the viral cycle and are the main targets of antiretroviral drugs (ARV) (Huff, 1991; Eron, 2000; El Safadi et al., 2007; Gu et al., 2020; Jóźwik et al., 2020). Molecular detection of Pol mutations associated with ARV resistance has enabled resistance monitoring and individualization of antiretroviral treatment (ART) regimens in HIV-positive subjects (Clarke, 2002). This approach is well extended in middle- and high-income countries, where clinicians often use online resistance interpretation algorithms, such as Stanford HIVdb Program^1^, to detect drug resistance mutations (DRM) in *pol* sequences and for HIV fast subtyping. HIV-1 *pol* diversity within HIV-1 variants is high and could impact ARV susceptibility (Holguín et al., 2006b). Surveillance of DRM in non-B subtypes and recombinants is essential (Holguín and Soriano, 2002; Holguín et al., 2004; Kantor, 2006; Llacer Delicado et al., 2016), as most studies focus on HIV-1 subtype B, more prevalent in Western Europe and the United States (Hemelaar et al., 2019).

The PR (99 aa) is responsible for processing the Gag and Gag–Pol precursors into mature Gag and Pol viral proteins by site-specific cleavage to produce the matrix, capsid, nucleocapsid, P1 and P2 spacer segments and P6 proteins of Gag, and the PR, RT, and IN proteins of Pol (Frankel and Young, 1998; Konvalinka et al., 2015). Variability in specific cleavage sites has been detected across HIV-1 groups, subtypes, and recombinants (Torrecilla et al., 2014). This could affect Gag and Pol proteins’ processing, viral budding, restore viral fitness, and influence the virological outcome of specific ARV (Goodenow et al., 2002; Myint et al., 2004; Holguín et al., 2006a; Dam et al., 2009). PR functions as a dimer with flexible flaps that close down on the active site upon substrate binding. This site resembles other aspartyl proteases with the conserved triad sequence Asp25-Thr26-Gly27 (Navia et al., 1989; Frankel and Young, 1998). There are five FDA-approved protease inhibitors currently recommended in the HHS HIV/AIDS medical practice guidelines (NIH FDA-Approved HIV Medicines, 2022).

The RT catalyzes RNA-dependent and DNA-dependent DNA polymerization reactions (Hu and Hughes, 2012). It is a heterodimer containing subunits p51 (440 aa) and p66 (560 aa), each with a polymerase domain composed of four subdomains (fingers, palm, thumb, and connection) and identical sequences, except for p66 additional RNase H domain (Rodgers et al., 1995). The polymerase active site contains the catalytic triad Asp110, Asp185, and Asp186, conserved in many polymerases (Frankel and Young, 1998). There are two classes of RT inhibitors: nucleoside RT inhibitors (NRTI) and non-nucleoside RT inhibitors (NNRTI), with a total of 10 FDA-approved RT inhibitors currently recommended in the HHS HIV/AIDS medical practice guidelines (NIH FDA-Approved HIV Medicines, 2022).

The IN (288 aa) catalyzes a series of reactions to integrate the viral genome into the host chromosome (Frankel and Young, 1998; Engelman and Singh, 2018). The N-terminal domain (aa 1–55) is dimeric and contains a zinc-binding site: His12, His16, Cys40, and Cys43 (Cai et al., 1997). The catalytic domain (aa 50–212) contains a D–D–E motif (Asp64, Asp116, and Glu152) conserved among integrases, essential for the processing and joining reactions (Dyda et al., 1994; Rice et al., 1996). Finally, the C-terminal domain has non-specific DNA-binding activity (Eijkelenboom et al., 1999). Integrase strand transfer inhibitors (INSTIs) are the most recently developed ARV drugs. There are three INSTIs approved by the FDA and currently recommended in the HHS HIV/AIDS medical practice guidelines (NIH FDA-Approved HIV Medicines, 2022). The three drugs bind to a common D–D–E motif in the IN catalytic domain, causing it to disengage (Sharma et al., 2014).

Since ARV development, more than 100 DRM have been described and classified as primary or secondary according to their effect on ARV efficacy (Shafer and Schapiro, 2008). During viral evolution, non-synonymous nucleotide mutations have been fixed along the viral genome, leading to aa changes; some of them are variant-specific (V-markers). Some V-markers can be related to drug resistance (R-markers) when fixed in positions associated with ARV resistance in the absence of antiretroviral therapy and may impact drug susceptibility and resistance pathways (Holguín et al., 2006b).

HIV Pol protein has an essential functional role in the viral cycle, being the main target for ARV and often used by clinicians to classify HIV-1 variants. The emergence of new HIV-1 variants and the spread of HIV-1 non-B subtypes and recombinants worldwide pose a challenge for the accuracy and efficiency of ARV, DRM detection, and fast subtyping online tools.

This descriptive study presents a thorough analysis of Pol diversity among HIV-1 variants circulating to date using ARV-naïve *pol* sequences available in Los Alamos National Laboratory HIV Sequence Database (LANL). We provide the aa conservation rate per residue within variants in PR, RT, and IN proteins. We also identify the V-markers and the R-markers across HIV-1 variants, analyzing the mean conservation of the consensus sequences of each HIV-1 variant and HIV-1 group in the three Pol proteins.

## Materials and Methods

**FIGURE 1:**
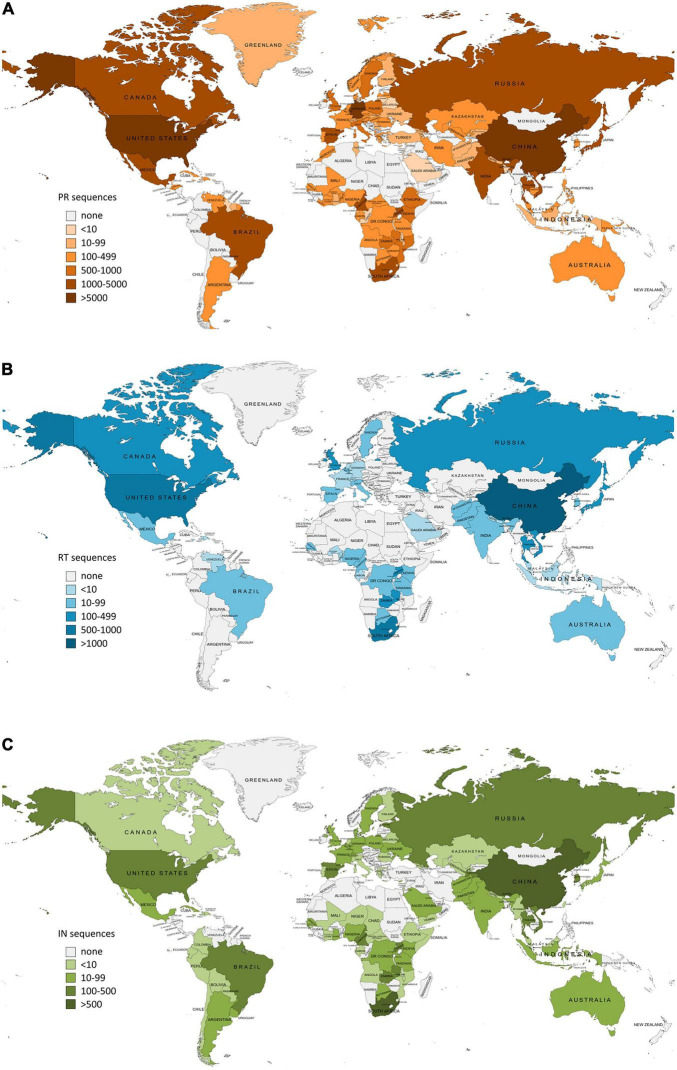
Number of HIV-1 Pol sequences per country included in this study as available in Los Alamos HIV sequence database (LANL) in January 2022. PR, protease; RT, reverse transcriptase; IN, integrase. **(A)** Protease sequences per country. Total LANL sequences: 59.733. **(B)** Reverse transcriptase sequences per country. Total LANL sequences: 6.437. **(C)** Integrase sequences per country. Total LANL sequences: 6.059. Eleven integrase sequences had no record of the country of origin.

**FIGURE 2:**
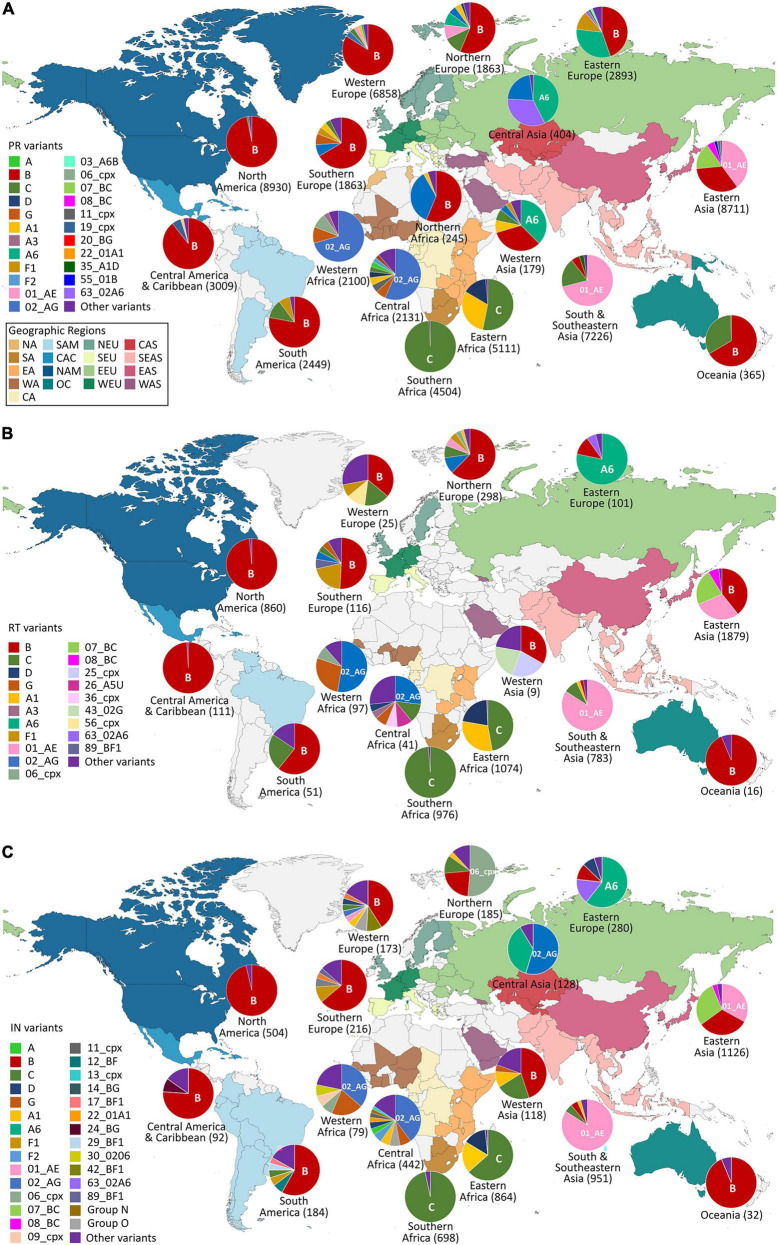
Geographic distribution by regions of HIV-1 Pol variants available in Los Alamos HIV sequence database (LANL) in January 2022. HIV-1 variant distribution within regions in PR **(A)**, RT **(B)**, and IN **(C)**. PR, Protease; RT, reverse transcriptase; IN, integrase. Countries are colored by regions according to the United Nations geoscheme (https://unstats.un.org). Geographic regions color code inside the box in **(A)**. Pie graphs show the percentage of the HIV-1 variants per region as available in LANL in January 2022 and the most frequent variant per region. The total number of available LANL sequences per region is in brackets beside the region name. NA, Northern Africa; SA, Southern Africa; EA, Eastern Africa; WA, Western Africa; CA, Central Africa; SAM, South America; CAC, Central America and The Caribbean; NAM, North America; OC, Oceania; NEU, Northern Europe; SEU, Southern Europe; EEU, Eastern Europe; WEU, Western Europe; CAS, Central Asia; SEAS, Southern and Southeastern Asia; EAS, Eastern Asia; WAS, Western Asia.

In January 2022, we downloaded from the LANL database^2^ all the available *pol* HIV-1 sequences from drug-naïve subjects carrying different HIV-1 variants (groups, subtypes, sub-subtypes, and CRFs), selecting the corresponding genome region (PR, RT, and IN). Before the downloading process, we selected only drug-naïve sequences and only one sequence per patient in the LANL (Los Alamos National Laboratory, 2022b) platform. We also considered as INSTI naïve all participants with IN sequences sampled before 2007, the year of marketing authorization of the first INSTI, raltegravir, and the start date of the first clinical study with an authorized INSTI both in Europe and in the United States. URF sequences and incomplete PR, RT, and IN sequences were not included in this study. The sequences were also sorted by country of origin and organized in geographic regions according to the United Nations geoscheme^3^ joining the regions of Central America and The Caribbean and the regions of Southern Asia and Southeastern Asia for practical purposes. The maps for Figures 1, 2 were created using MapChart^4^.

A sequence analysis was performed with an in-house bioinformatics tool (EpiMolBio) previously designed and used in our laboratory for HIV genetic variability analysis and recently updated for SARS-CoV-2 sequences study (Burgos et al., 2019; Troyano-Hernáez et al., 2019, 2020, 2021a,b,^2022^). This tool is programmed in JAVA OpenJDK version 11.0.9.1 using IDE NetBeans version 12.2. Among other functions, this tool calculates the conservation of a sequence set compared with a reference sequence and the rate of aa changes for each position within the studied protein. Furthermore, it can infer a consensus from a group of sequences or previously calculated consensuses considering the total number of sequences and the frequency of any specific aa residue per position, avoiding the overestimation of polymorphisms present in variants with a small number of available sequences. We used HIV-1 reference sequence HXB2 (NCBI accession number K03455.1) for the sequences’ alignment and EpiMolBio functions that required a reference sequence, such as conservation analysis and V-marker detection.

We inferred the PR, RT, and IN consensus sequence for HIV-1, each HIV-1 group (M, N, O, and P), and each HIV-1 group M variant (subtype, sub-subtype, CRF) using all downloaded LANL sequences. Group M consensus was generated from the consensus of group M subtypes, sub-subtypes, and CRF. HIV-1 consensus was inferred considering the consensuses of the four groups (M, N, O, and P). We calculated the mean aa conservation of group M and HIV-1 consensus sequences for the three Pol proteins and the variability of the residues involved in binding or catalytic sites. We also studied the average aa conservation of the PR, RT, and IN group M variants with >5 available sequences compared with HXB2 HIV-1 reference sequence.

We identified the presence of single variant markers or V-markers, defined as the natural aa changes specific for each variant and present in >75% of the sequence set for a given position in variants with >5 sequences available in LANL, to avoid biases due to a low number of sequences. We considered as R-markers, the V-markers coinciding with major or minor DRM to the four main ARV families (PI, NRTI, NNRTI, and INSTI) according to the updated version of two sources: Stanford HIV Drug Resistance Database v9.0^5^ and IAS-USA 2019 (Wensing et al., 2019). The sub-classification of DRM into major or minor DRM was done following the Stanford Database 9.0 criteria, which considers the effect on *in vitro* drug susceptibility, the frequency among patients with virological failure, the presence among untreated persons, and the location of the mutation within the 3D structure protein. Deletions and insertions were not included in this study.

In the *pol* variants with >5 available sequences, besides detecting V- and R-markers, we also checked for the presence of drug resistance mutations present in the WHO 2009 list for transmitted mutations or TDR (Bennett et al., 2009) and of major and minor DRM present in at least 25% of the sequences for each variant. To study the effect of these DRM and R-markers on ARV susceptibility, we analyzed them with the online resistance interpretation algorithm Stanford HIVdb Program v9.0^6^, which infers susceptibility to 25 ARV from PI, NRTI, NNRTI, and INSTI drug families.

We calculated the Wu–Kabat protein variability coefficient (WK) for group M using all available PR, RT, and IN sequences belonging to this group. WK coefficient allows studying the susceptibility of an aa position to evolutionary replacements (Kabat et al., 1977). It was calculated using the following formula: variability = *N* × *k*/*n*, where *N* is the number of sequences in the alignment, *k* is the number of different amino acids at a given position, and *n* is the absolute frequency of the most common amino acid at that position. Therefore, a WK of 1 indicates the same aa was found for that position in all the sequence set, whereas a WK >1 indicates the relative variability of the respective site, with greater diversity as the WK value increases.

## Results

### Analyzed Pol Sequences and Inferred Consensus Sequences

A total of 59,733 PR (32,745 group M non-B subtypes and CRF or non-B variants), 6,437 RT (4,393 non-B variants), and 6,059 IN (4,552 non-B variants) sequences were included in this study (Supplementary Table 1). Subtypes with the greatest sequence representation in group M were subtype B (45.1% in PR, 31.6% in RT, and 23.8% in IN), followed by subtype C (16.6% in PR, 25.0% in RT, and 22.3% in IN). The most represented CRF was recombinant 01_AE (15.2% in PR, 19.0% in RT, and 19.7% in IN).

The country of origin of the LANL available sequences for each Pol protein is illustrated in Figure 1 (complete information in Supplementary Table 2). The geographic distribution by regions of the HIV-1 variants with available *pol* sequence in LANL is illustrated in Figure 2 (described in Supplementary Table 3). PR sequences showed the highest diversity regarding the number of countries of origin of the sequenced samples (118 countries) compared with RT (50 countries) and IN (85 countries). In the three Pol proteins, China was the country that contributed the most sequences to the LANL database (11% in PR, 22% in RT, and 16% in IN), followed by South Africa in RT (15%) and IN (11%), and the United States in PR (10%), which was in third place in RT (11%) and IN (8%). Thus, the countries present in each geographic region were not homogeneous in the three Pol proteins, and some geographic regions presented different HIV-1 main variants between Pol proteins. However, subtype B was the main variant for PR, RT, and IN in America, Western and Southern Europe, and Oceania, while there was a greater percentage of subtype A6 in Eastern Europe, CRF 01_AE in Southern, Southeastern, and Eastern Asia, and subtype C in Southern and Western Africa. The regions with more variant diversity in the three proteins were Central and Western Africa (Figure 2).

**FIGURE 3:**
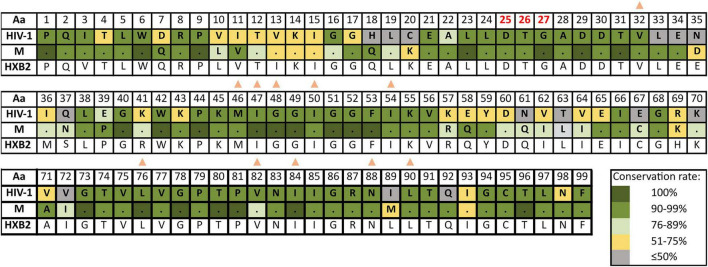
Amino acid conservation rate along PR in HIV-1 and group M consensus. aa, amino acid; M, group M consensus. PR, protease (99 aa). Dots in group M represent the same aa as in HIV-1 consensus for that position. HXB2 reference sequence is described below the groups for further guidance. Colors represent the conservation rate. Residues of PR active site (triad Asp25-Thr26-Gly27, conserved among aspartyl proteases) are highlighted in red font. Orange triangles indicate positions where major DRM to PI are located according to Stanford v9.0 (Release Notes - HIV Drug Resistance Database, 2020) and summarized in https://cms.hivdb.org/prod/downloads/resistance-mutation-handout/resistance-mutation-handout.pdf. Aa code: A, alanine; C, cysteine; D, aspartic acid; E, glutamic acid; F, phenylalanine; G, glycine; H, histidine; I, isoleucine; K, lysine; L, leucine; M, methionine; N, asparagine; P, proline; Q, glutamine; R, arginine; S, serine; T, threonine; V, valine; W, tryptophan; Y, tyrosine.

**FIGURE 4:**
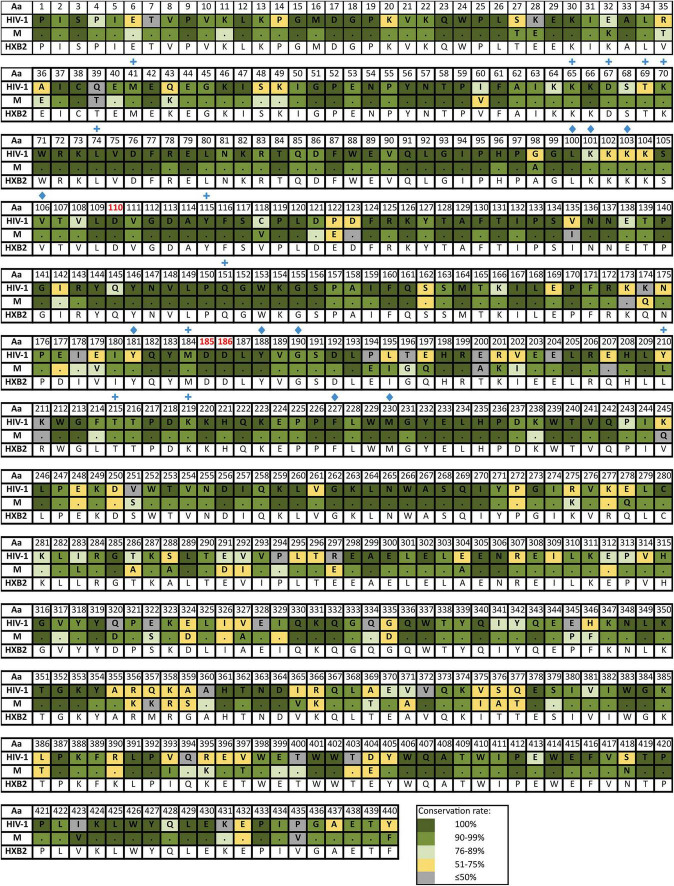
Amino acid conservation rate along RT in HIV-1 and group M consensus. Aa, amino acid; M, group M consensus. RT, reverse transcriptase (440 aa). Dots in group M represent the same aa as in HIV-1 consensus for that position. HXB2 reference sequence is described below the groups for further guidance. Colors represent the conservation rate. Residues of the catalytic triad (Asp110, Asp185, and Asp186) are highlighted in red font. Blue crosses and blue diamonds indicate positions where DRM to NRTI and to NNRTI, respectively, are located according to Stanford v9.0 (Release Notes - HIV Drug Resistance Database, 2020). Aa code according to Figure 3.

**FIGURE 5:**
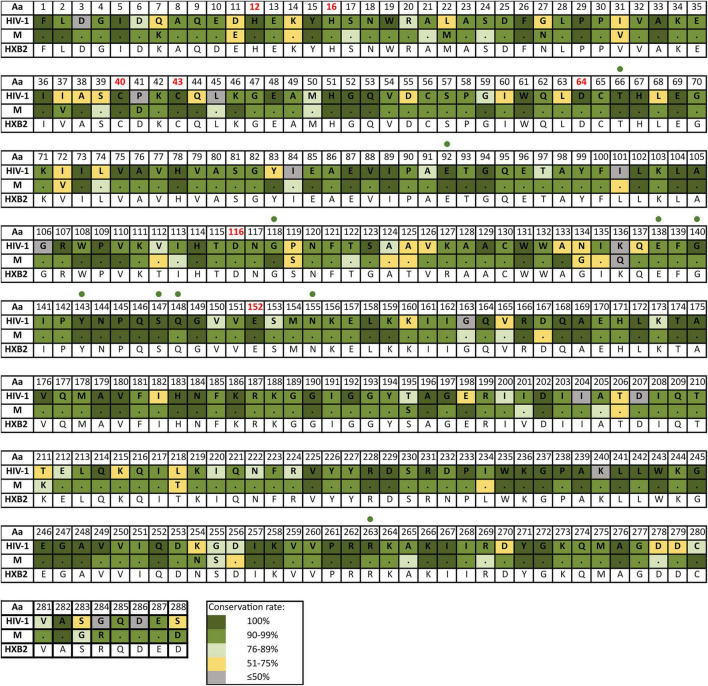
Amino acid conservation rate along IN in HIV-1 and group M consensus. Aa, amino acid; M, group M consensus. IN, integrase (288 aa). Dots in group M represent the same aa as in HIV-1 consensus for that position. HXB2 reference sequence is described below the groups for further guidance. Colors represent the conservation rate. Residues of the zinc-binding site (His12, His16, Cys40, and Cys43) and the D–D–E motif of the catalytic domain (Asp64, Asp116, and Glu152) are highlighted in red font. Green circles indicate positions where major INSTIs DRM are located according to Stanford v9.0 (Release Notes - HIV Drug Resistance Database, 2020). Aa code according to Figure 3.

Supplementary Table 4 reports the inferred consensus sequences for HIV-1, each HIV-1 group and group M variant in the three Pol proteins, showing the most frequent aa found per residue. Group M consensus was inferred using 84 variants’ consensus in PR, 52 in RT, and 86 in IN. HIV-1 consensus and group M consensus sequences of PR, RT, and IN are displayed in Figures 3–5, respectively. The percentage of conservation of the most prevalent aa in each residue is indicated with a color code: dark green (100%), green (≥90–<100%), light green (>75–<90%), yellow (>50–≤75%), and gray (≤50%). The HXB2 reference sequence was included for further guidance. These figures also indicate the positions where major DRM to the four main drug families are located according to Stanford v9.0 and the location of PR, RT, and IN catalytic sites.

The mean aa conservation of HIV-1 and group M consensus sequences was 82.60 and 93.11% in PR, 88.81 and 94.07% in RT, and 90.98 and 96.02% in IN, respectively. Table 1 describes the variability of PR, RT, and IN active sites and the mean residue variability in HIV-1 consensus for each protein. All the residues involved in binding or catalytic sites showed a variability below 0.5%, indicating a conservation over 95%. The mean aa conservation percentage in Pol residues of PR, RT, and IN is shown in Figures 3–5, respectively.

**TABLE 1:** Variability in protease (PR), retrotranscriptase (RT), and integrase (IN) active sites.

Protein	Sites		Variability
PR (99 aa)	Complete PR		17.40%*
	Active site triad	Asp25	0.11%
		Thr26	0.04%
		Gly27	0.01%
RT (440 aa)	Complete RT		11.19%*
	Catalytic triad	Asp110	0.03%
		Asp185	0.04%
		Asp186	0.06%
IN (288 aa)	Complete IN		9.02%*
	Zinc-binding site	His12	0.08%
		His16	0.15%
		Cys40	0.13%
		Cys43	0.05%
	D–D–E motif	Asp64	0.07%
		Asp116	0.08%
		Glu152	0.23%

*PR, protease; RT, reverse transcriptase; IN, integrase; with an asterisk, mean residue variability for each protein calculated from their respective HIV-1 consensus.*

### Protease, Retrotranscriptase, and Integrase aa Conservation Across HIV-1 M Variants

**FIGURE 6:**
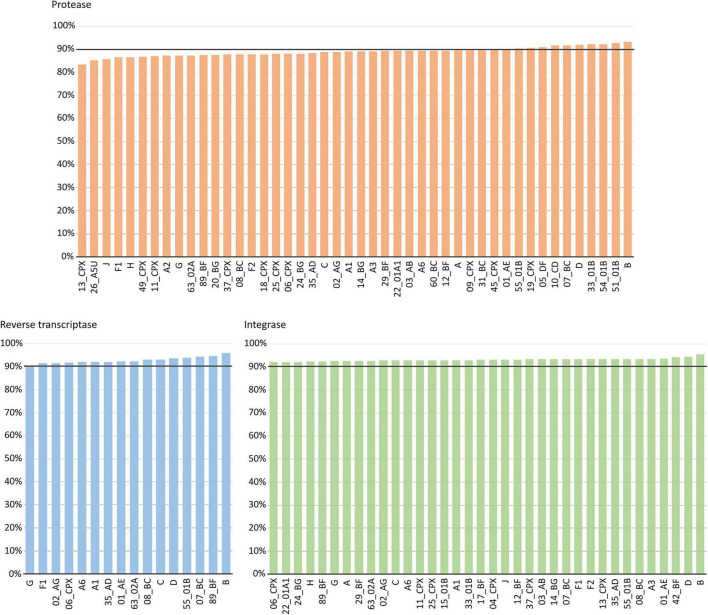
Percentage of aa conservation of PR, RT, and IN across the HIV-1 group M variants with >5 sequences at LANL. *X*-axis: HIV-1 group M variants with >5 available sequences at LANL (46 in PR, 16 in RT, and 36 in IN). *Y*-axis: conservation rate for each variant included in this analysis. The horizontal line represents 90% conservation.

We included in this analysis 46 variants in PR, 16 in RT, and 36 in IN, all with >5 sequences in LANL. Subtype B, the variant with the highest number of Pol sequences at LANL, was the most conserved variant in the three proteins (93.22% in PR, 96.02% in RT, and 92.09% in IN). The most conserved CRFs were 51_01B in PR (92.80%), 89_BF in RT (94.62%), and 42_BF in IN (94.31%). The least conserved variants were CRF13_cpx in PR (83.5%), subtype G in RT (90.44%), and CRF06_cpx in IN (92.09%). In RT and IN, all the variants had a conservation >90%, whereas, in PR, only 24% of the variants with >5 sequences had a conservation above 90% (Figure 6).

### V-Markers, R-Markers, and Other Drug Resistance Mutations

Among the variants with >5 available sequences, we found a total of 106 unique single V-markers and 8 R-markers present in >75% sequences (>75% conservation in their respective variants) across PR (Table 2), RT (Table 3), and IN (Table 4) variants. The analysis was performed in group O and 46 group M variants (7 subtypes, 6 sub-subtypes, and 33 CRF) of PR; in 16 group M variants (4 subtypes, 3 sub-subtypes, and 9 CRF) of RT; and in groups N and O, and 36 group M variants (7 subtypes, 5 sub-subtypes, and 24 CRF) of IN.

**TABLE 2:** Single V-markers and R-markers in protease found across HIV-1 variants with >5 LANL sequences.

HIV-1 variant	Countries	V-markers and R-markers (bold red)
Group O (24)	Belgium (2), Cameroon (12), Senegal (3), Spain (3), United Kingdom (2), United States (2)	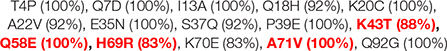
Subtype J (20)	Republic of Angola (2), The Democratic Republic of the Congo (2), Central African Republic (3), Congo (1), Cameroon (7), Gabon (1), Spain (1), Senegal (1), Belgium (2)	Q61E (83%)
CRF08_BC (431)	China (430), India (1)	T12S (87%)
CRF13_cpx (42)	Belgium (2), Burkina Faso (1), Cameroon (22), Central African Republic (5), Germany (5), Greenland (1), Poland (1), Spain (2), The Democratic Republic of the Congo (3)	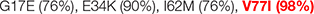
CRF19_cpx (178)	Cuba (172), Spain (4), Tunisia (1), United Kingdom (1)	H69Q (75%)
CRF35_A1D (216)	Afghanistan (9), China (1), Iran (205), Romania (1)	L19Q (76%)
CRF49_cpx (10)	Botswana (1), Gambia (3), Germany (4), Nigeria (1), Senegal (1)	D60N (90%), Q61D (90%), I66V (80%)
CRF51_01B (8)	Singapore (8)	L63S (100%)
CRF60_BC (25)	Brazil (1), Germany (2), Italy (22)	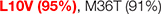
CRF63_02A6 (193)	Kyrgyzstan (2), Russian Federation (59), Uzbekistan (132)	K14R (79%)
CRF89_BF1 (22)	Argentina (1), Spain (20), Sweden (1)	T12E (91%)

*In brackets, number of sequences in variant or country and conservation percentage in markers; in bold red font, V-markers that are R-markers. None of the R-markers corresponded to major DRM to PI according to Stanfordv 9.0.*

**TABLE 3:** Single V-markers and R-markers in reverse transcriptase found across HIV-1 variants with >5 LANL sequences.

HIV-1 variant	Countries	V-markers and R-markers (bold red)
Sub-subtype A6 (85)	United Kingdom (4), Georgia (1), Italy (1), Russian Federation (79)	K11T (85%), E36D (76%)
Subtype C (1607)	Belgium (4), Brazil (12), Botswana (30), The Democratic Republic of the Congo (4), China (11), Spain (5), United Kingdom (14), Georgia (1), Equatorial Guinea (1), India (65), Kenya (4), Nigeria (1), Nepal (2), Pakistan (1), Rwanda (3), Sweden (9), Senegal (2), Thailand (1), United Republic of Tanzania (37), Uganda (7), United States (5), South Africa (933), Zambia (455)	T39E (78%)
Subtype D (238)	The Democratic Republic of the Congo (2), United Kingdom (2), Kenya (1), South Korea (1), Nigeria (1), United Republic of Tanzania (1), Uganda (229), United States (1)	L282C (91%), P345Q (89%), T377Q (92%), S379C (82%)
Sub-subtype F1 (41)	Germany (1), Spain (22), France (1), United Kingdom (14), Italy (2), United States (1)	D123E (88%), I178L (85%)
Subtype G (40)	The Democratic Republic of the Congo (2), Cameroon (1), Spain (5), United Kingdom (2), Kenya (1), Nigeria (27), Russian Federation (1), South Africa (1)	M357R (93%), Q394R (90%), T400V (83%), F440Y (93%)
CRF01_AE (1225)	Afghanistan (1), China (547), Sweden (3), Thailand (221), United Kingdom (13), United States (3), Viet Nam (437)	V245E (91%)
CRF02_AG (110)	Belgium (1), Benin (2), Cameroon (5), China (2), Spain (5), Gabon (1), United Kingdom (29), Equatorial Guinea (5), Italy (1), South Korea (1), Mexico (1), Nigeria (33), Pakistan (3), Russian Federation (1), Sweden (2), Senegal (9), Togo (7), Thailand (1), United States (1)	S162A (96%)
CRF06_cpx (19)	Burkina Faso (3), China (1), United Kingdom (10), Nigeria (4), Senegal (1)	F346H (84%), R358K (89%)
CRF08_BC (129)	China (129)	E53D (98%), D324E (86%)
CRF35_A1D (9)	Afghanistan (9)	L283I (78%)
CRF55_01B (11)	China (11)	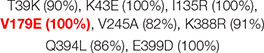
CRF89_BF (7)	Spain (7)	Q394L (86%), E399D (100%)

*In brackets, number of sequences in variant or country and conservation percentage in markers; in bold red font, V-markers that are R-markers. None of the R-markers corresponded to major DRM to NRTI or NNRTI according to Stanford v9.0.*

**TABLE 4:** Single V-markers and R-markers in integrase found across HIV-1 variants with >5 LANL sequences.

HIV-1 variant	Countries	V-markers and R-markers (bold red)
Group N (12)	Cameroon (11), France (1)	D55N (100%), V165I (92%), K215T (100%), T218L (100%), I220V (83%), D279G (100%)
Group O (50)	Cameroon (30), Belgium (1), France (13), United States (3), Senegal (3)	D3E (90%), K7Q (100%), M22L (100%), N27G (100%), D41P (100%), Q44H (98%), L45I (88%), G59E (96%), Y83F (98%), G106A (100%), V126M (100%), Q137H (94%), S153A (96%), K160S (98%), G163Q (90%), I182V (90%), I204L (100%), D207Q (80%), K211T (100%), K240Q (100%), N254K (98%), C280S (86%), V281M (84%), D286T (92%), D288S (94%)
Subtype C (1353)	Cameroon (1), Ethiopia (2), Kenya (6), Malawi (1), Mozambique (1), Rwanda (3), Somalia (1), United Republic of Tanzania (57), Uganda (7), Zambia (471), Poland (1), Belgium (7), Denmark (1), Spain (5), Sweden (20), United States (2), Botswana (52), South Africa (623), Argentina (1), Brazil (7), Uruguay (1), Senegal (2), China (4), Cyprus (4), Georgia (1), India (43), Myanmar (1), Israel (5), Nepal (3), Pakistan (1), Saudi Arabia (14), Tajikistan (2), Thailand (1), Unknown (1), Yemen (1)	D25E (79%)
Subtype H (11)	Belgium (3), Cameroon (1), Central African Republic (2), The Democratic Republic of the Congo (4), United Kingdom (1)	N222K (91%)
Subtype J (10)	Republic of Angola (1), Belgium (3), Cameroon (1), The Democratic Republic of the Congo (3), Sweden (2)	Y99F (80%)
CRF03_A6B (8)	Belarus (1), Russian Federation (3), Tajikistan (4)	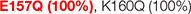
CRF06_cpx (103)	Australia (1), Cameroon (2), Estonia (95), Ghana (1), Mali (2), Russian Federation (1), Senegal (1)	L63I (91%)
CRF07_BC (324)	China (321), South Korea (1), Taiwan (1), Viet Nam (1)	I84M (91%)
CRF08_BC (36)	China (36)	K211R (92%)
CRF22_01A1 (14)	Cameroon (14)	A23V (100%)
CRF33_01B (6)	Indonesia (2), Malaysia (4)	L63V (83%)
CRF35_A1D (13)	Afghanistan (13)	I60M (100%), V126F (100%), G134S (77%)
CRF42_BF1 (17)	Luxembourg (17)	L28I (100%), S39C (100%), G163E (100%)

*In brackets, number of sequences in variant or country and conservation percentage in markers; in bold red font, V-markers that are R-markers. None of the R-markers corresponded to major DRM to INSTI according to Stanford v9.0.*

We detected 31 V-markers in PR (6 of them being R-markers), 28 V-markers in RT (1 R-marker), and 47 in IN (1 R-marker). None of the R-markers corresponded to major DRM, being all of them minor DRM according to Stanfordv 9.0. No V-markers were observed in the PR active site (Asp25, Thr26, and Gly27), in the RT catalytic triad (Asp110, Asp185, and Asp186), the IN zinc-binding site (His12, His16, Cys40, and Cys43), or the D–D–E motif (Asp64, Asp116, and Glu152) of the IN catalytic domain.

The 31 V-markers in PR were present in 11 variants (9 CRF, subtype J, and group O), being group O the variant with most V-markers: 15 (Table 2). Six (19.3%) of the 31 PR V-markers corresponded to R-markers: Four were detected in group O (K43T/Q58E/H69R/A71V) and two in group M complex recombinants CRF13_cpx (V77I) and CRF60_BC (L10V). The R-markers in group O were present in 83.3% (H69R), 87.5% (K43T), and 100% (Q58E, A71V) of this group’s PR sequences. L10V was found in 95.5% of CRF60_BC PR sequences and V77I in 96.6% of CRF13_cpx sequences. In RT, we detected a total of 28 V-markers in 12 group M variants (5 subtypes and 7 CRF). Only one (3.6%) of them was an R-marker: V179E, found in 100% of CRF55_01B RT sequences (Table 3). The largest number of V-markers was found in IN: 47 total V-markers in two non-M groups and 11 group M variants (3 subtypes and 8 CRF) (Table 4). Group O presented most V-markers (25/47, 53.2%). The only R-marker detected was E157Q in 100% of CRF03_A6B sequences.

No TDR present in the WHO list or major DRM to PI, NNRTI, NRTI, or INSTI were found in ≥25% of sequences belonging to PR, RT, or IN variants with >5 available sequences. However, we found seven minor DRM in the three Pol proteins. Two minor DRM to PI were detected in PR: Q58E and K43T, coinciding with two of group O R-markers in PR (Table 2). Another two minor DRM to NNRTI were found in RT: V179E in CRF06_cpx (29.4% sequences), coinciding with the only R-marker found in RT in CRF55_01B (Table 2), and V106I in sub-subtype F1 (42.5%). We found one minor DRM to NRTI, A62V, present in 47% of RT sequences of sub-subtype A6. Finally, two minor DRM to INSTI were detected in IN: G163R in CRF17_BF1 (28.6% sequences) and CRF89_BF1 (50%), and M50I in group O (49%), subtype A (60%), subtype C (48%), CRFs 11_cpx (31.2%), CRF22_01A1 (78.6%), and CRF63_02A6 (76.6%).

### Wu–Kabat Pol Variability Coefficient in Protease, Retrotranscriptase, and Integrase Group M

**FIGURE 7:**
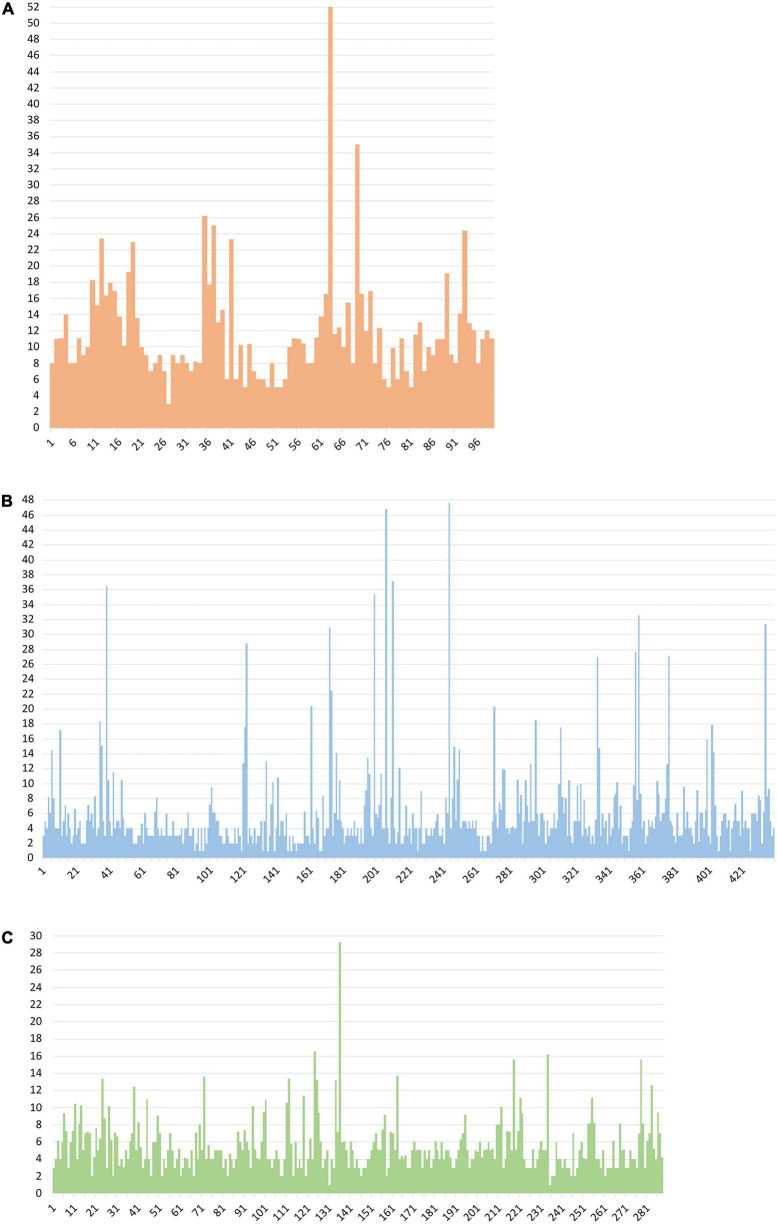
Wu–Kabat variability coefficient plot of PR, RT, and IN group M sequences. **(A)** Wu–Kabat variability coefficient plot of PR (99 aa). **(B)** Wu–Kabat variability coefficient plot of RT (440 aa). **(C)** Wu–Kabat variability coefficient plot of IN (288 aa). *X*-axis, amino acid position; *Y*-axis, WK variability coefficient.

Figure 7 describes the group M variability WK coefficient plot in the three *pol* proteins using all available LANL sequences for this group (26,988 PR, 2,044 RT, and 1,507 IN), including 84/52/86 HIV-1 group M variants in PR/RT/IN: 9/5/9 subtypes, 8/6/7 sub-subtypes, and 67/41/70 CRF. The WK values for each residue and studied protein are described in Supplementary Table 5.

**FIGURE 8:**
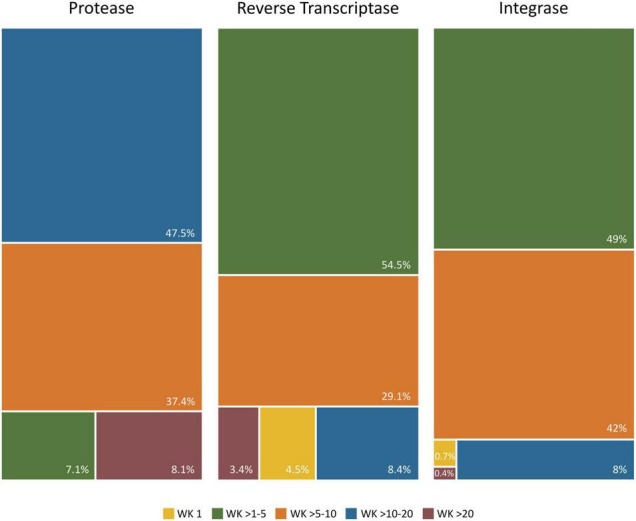
Proportion of Wu–Kabat variability coefficient values in PR, RT, and IN residues. Each box represents the proportion of residues within each protein that present a Wu–Kabat coefficient value within the range indicated beneath the figure and colored accordingly. Protease (99 aa), reverse transcriptase (440 aa), integrase (288 aa). WK, WK variability coefficient.

The median variability coefficient in PR group M sequences was 10.26. The highest WK coefficient was 52 in residue 63, followed by WK 35 in site 69 (Figure 7A). The lowest WK was 3 in site 27, part of the triad Asp25, Thr26, and Gly27 in the PR active site. The other two residues of this triad (Asp25 and Thr26) presented a WK of 9 and 7, respectively. None of the 99 residues along PR were completely conserved (WK 1). Most PR residues had a WK between 10 and 20 (Figure 8).

The median WK along RT in group M sequences was 4.03. Site 245 presented the highest WK coefficient (WK 47.61), followed by site 207 (WK 46.84) and site 211 (WK 37.2) (Figure 7B). The smallest WK was 1, present in 20 of the 440 RT residues. The RT catalytic triad Asp110, Asp185, and Asp186 had a WK of 2, 3, and 4, respectively. Most RT residues had a WK between 1 and 5 (Figure 8).

IN median variability coefficient in group M sequences was 5. The highest WK coefficient was 29.35, located in residue 136, followed by site 125 (WK 16.6) and site 234 (WK 16.22) (Figure 7C). The smallest WK was 1, present in sites 131 and 235. The residues of the IN zinc-binding site, namely, His12, His16, and Cys40, showed a WK coefficient of 4, 7, and 5, respectively. The IN catalytic domain’s Asp64, Asp116, and Glu152 motif presented a WK of 4, 3, and 6, respectively. Most IN residues had a WK between 1 and 5 (Figure 8).

## Discussion

This descriptive study analyzes the Pol diversity among HIV-1 variants, providing the aa conservation rate per residue and HIV-1 variant in PR, RT, and IN proteins. A better understanding of HIV variability is important, since it has been reported that HIV-1 transmissibility, replication, and disease progression can differ between HIV-1 variants (Renjifo et al., 2004; Baeten et al., 2007; Bennett et al., 2009; Ng et al., 2014). The HIV-1 Pol proteins PR, RT, and IN are essential for viral replication and are the main targets of ARV (Huff, 1991; Eron, 2000; El Safadi et al., 2007; Gu et al., 2020; Jóźwik et al., 2020). Since Pol variability can impact ARV monitoring and efficacy, conservation studies must consider all circulating HIV-1 variants worldwide.

The consensus sequences of HIV proteins and their conservation studies allow a better understanding of structural, functional, and immunogenic potential differences across HIV-1 groups, subtypes, sub-subtypes, and recombinants and have been previously analyzed in other HIV-1 proteins (Li et al., 2013; Sliepen et al., 2019; Zhang et al., 2021). A recent work by Linchangco et al. reconstructed 90 HIV-1 subtype and CRF consensus sequences from 3,470 full HIV genomes downloaded from LANL (Linchangco et al., 2021). Our study updates and expands the knowledge regarding HIV Pol variability, including 59,733 PR, 6,437 RT, and 6,059 IN sequences from more than 100 different variants, including all the currently available HIV-1 groups, subtypes, and CRF in LANL. Moreover, Supplementary Table 4 summarizes the aa conservation in each Pol protein and each variant to help identify the conservation or consensus aa in any Pol residue and HIV-1 variant of interest.

The sequences were processed by an in-house bioinformatics tool (EpiMolBio) developed for HIV and SARS-CoV-2 variability analysis. In the most extensive panel of HIV-1 variants analyzed to date, we have also identified the natural polymorphisms that can be considered as genetic markers of each HIV-1 variant (V-markers) and those that correspond to major or minor DRM (R-markers) across HIV-1 groups, and group M subtypes and recombinants. We also present the consensus PR, RT, and IN sequences for HIV-1, HIV-1 groups, and variants, and the Wu–Kabat variability coefficient for group M in the three studied Pol proteins. This information is helpful to improve the understanding of structural, functional, and immunogenic differences across HIV-1 groups, subtypes, sub-subtypes, and recombinants and their impact on drug susceptibility and resistance pathways (Nagata et al., 2017; Sliepen et al., 2019; Zhang et al., 2021).

In previous studies, the variability in Pol proteins was low but slightly higher in PR compared with RT and IN (Turner et al., 2004; Rhee et al., 2016). In this study, the mean aa conservation of group M consensus sequences was high (>90%) in the three studied proteins, being slightly lower for PR (93% vs. 94% in RT and 96% in IN). As expected, HIV-1 consensus sequences were slightly less conserved (>80%) as non-M groups were included in the consensus. Still, the conservation rate followed the same order as in group M consensus, with less conservation in PR (83% vs. 88% in RT and 96% in IN). We also observed a low variability (below 0.5%) in the residues involved in binding or catalytic sites after testing all the available Pol sequences, highlighting the fragility of these important functional sites (Table 1).

HIV-1 variants have different global prevalence (Hemelaar et al., 2019) and levels of HIV-1 genetic diversity (Abecasis et al., 2009). HIV-1 group M subtype C is the most prevalent variant in the ongoing HIV pandemic, causing around 50% of worldwide infections (Hemelaar et al., 2019). In addition, subtype C is the most prevalent variant in Southern Africa and India; subtype A in some countries of Eastern Africa, Russia, and Eastern Europe; subtype B in the rest of Europe, the Americas, and Oceania; CRF01_AE in Asia; and CRF02_AG in Western Africa (Bbosa et al., 2019). However, HIV genomic sequencing is more widespread in economically developed nations, which explains that in our Pol dataset the most represented HIV-1 variant was subtype B, despite the fact that this variant only causes around 12% of the 38 million infections globally (Hemelaar et al., 2019), followed by the most abundant variant subtype C and recombinant CRF01_AE with the highest number of sequences belonging to China and the United States, according to the sequence availability in LANL. The main limitation in this study is the low number of sequences available in LANL for some non-B subtypes and CRF (Hemelaar et al., 2019), due to their low prevalence in the pandemic or because they are circulating in areas with none or scarce HIV sequencing.

Across group M variants with >5 available sequences in LANL, subtype B was the most conserved variant (>92%) in the three Pol proteins as expected, since the reference strain for the alignments was the subtype B HXB2 isolate. Again, PR showed slightly greater variability: While in RT and IN all the included variants had a conservation >90%, in PR only 24% of the variants were conserved in >90% of their sequences.

The Wu–Kabat protein variability coefficient (WK) was analyzed in PR, RT, and IN group M to study the susceptibility of each aa position to evolutionary replacements. The median variability coefficient in PR (WK10) was higher than that in IN (WK5) and RT (WK4). All 99 PR residues presented some degree of variability as none had a coefficient of 1. PR also presented the site with the highest WK value (52 in residue 63). Most IN (92%) and RT (88%) sites showed a WK below 10, while almost half (48%) of PR residues had a WK between 11 and 20, being the Pol protein with more sites prone to evolutionary replacements.

Although similar mutations occur in subtype B and non-subtype-B viruses and drug resistance evolution is comparable in both groups, subtype-specific mutation rates have been identified, with differences that could affect genotypic interpretation and DRM monitoring (Kantor et al., 2005; Kantor, 2006; Yebra et al., 2010). We found 31 total V-markers in PR, 28 V-markers in RT, and 47 in IN. Only eight were R-markers; none considered major DRM, being minor DRM with low impact in ARV susceptibility. In a previous study on HIV-2 variability (Troyano-Hernáez et al., 2021a), the R-markers corresponding to DRM to PI, NRTI, and INSTIs appeared not to have a significant impact on ARV susceptibility as well. However, HIV-2 presents natural polymorphisms related to drug resistance that make it naturally resistant to NNRTI, certain PI, and fusion inhibitor enfuvirtide (Tuaillon et al., 2004; Desbois et al., 2008; Menéndez-Arias and Álvarez, 2014).

Six R-markers were found in PR (K43T/Q58E/H69R/A71V in group O, V77I in CRF13_cpx, and L10V in CRF60_BC), one in RT (V179E in CRF55_01B), and one in IN (E157Q in CRF03_A6B). None of the R-markers in PR’s group O conferred intermediate or high-level resistance to PI alone or combined. K43T and Q58E are accessory non-polymorphic mutations that confer potential or low-level resistance to nelfinavir (NFV) and tipranavir/ritonavir (TPV/r) and other PIs (Rhee et al., 2003, 2010; Baxter et al., 2006; Bennett et al., 2009). H69R is a minor mutation affecting TPV/r according to IAS (Wensing et al., 2019) (not included in Stanford). A71V is a polymorphic accessory mutation associated with an increase of viral replication in the presence of other PI resistance mutations (Nijhuis et al., 1999; Rhee et al., 2003). V77I was present in 96.6% of CRF13_cpx PR sequences and is considered a minor mutation affecting indinavir/ritonavir according to IAS2019 (Wensing et al., 2019) (not included in Stanford). L10V was found in 95.5% of CRF60_BC PR sequences, being a polymorphic accessory mutation that may reduce PI susceptibility or increase the replication of viruses containing PI resistance mutations (Stanford University, 2022). Regarding the R-marker found in RT, V179E is a DRM to NNRTI, considered a non-polymorphic accessory mutation associated with potential low-level resistance to efavirenz (EFV), etravirine (ETR), nevirapine (NVP), and rilpivirine (RPV) (Rhee et al., 2003; Tambuyzer et al., 2009). As for the R-marker present in IN, E157Q is an accessory mutation with little effect by itself on the response to INSTI therapy, conferring potential low-level resistance to elvitegravir (EVG) and raltegravir (RAL) (Anstett et al., 2016; Stanford University, 2022; Charpentier et al., 2018).

Similarly, when analyzing the seven DRM found in ≥25% of the sequences in variants with >5 available PR, RT, or IN sequences, none corresponded to major DRM. Three were R-markers: the previously described accessory DRM to PI, Q58E and K43T, and the accessory DRM to NNRTI V179E. In RT, we identified another accessory DRM to NNRTI, V106I, in sub-subtype F1. This mutation is present in 1–2% of naïve patients and contributes to reduced NNRTI susceptibility combined with other mutations, such as V179D, not found in the available F1 sequences (Rhee et al., 2003; Gatanaga et al., 2010). Alone it has little effect on NNRTI susceptibility conferring potential low-level resistance to doravirine (DOR), ETR, NVP, and RPV (Release Notes—HIV Drug Resistance Database). A62V (sub-subtype A6) is an accessory mutation that often occurs together with the multi-NRTI resistance mutations K65R or Q151M (Svarovskaia et al., 2008). However, these mutations were not found in this subtype among our sequence sets. A62V is widespread in subtype A viruses belonging to the former Soviet Union countries but is otherwise non-polymorphic (Carr et al., 2005). Two accessory DRM to INSTI were detected in IN: G163R, found in two CRFs, and M50I, in six variants. G163R is a non-polymorphic mutation that confers low-level resistance to EVG and RAL and usually appears in combination with N155H (Gatell et al., 2010; Stanford University, 2022), not found in these CRFs. M50I is a polymorphic mutation that may reduce dolutegravir (DTG) susceptibility in combination with R263K (Wares et al., 2014), absent in all the variants carrying M50I.

We present a thorough descriptive analysis of Pol variability among all HIV-1 variants circulating to date. The relatively high aa conservation observed in Pol proteins across HIV-1 variants highlights their critical role in the viral cycle. The variant-specific polymorphisms (V-markers) found in Pol presented little or no predicted impact on clinical ARV efficacy. Our data support previous studies reporting limited evidence of associations between HIV-1 subtypes and treatment failure (Rockstroh et al., 2011; Poon et al., 2019). However, it has been reported that some natural polymorphisms in Pol can promote alternative resistance pathways (Kantor and Katzenstein, 2003; Holguín et al., 2006b; Sanches et al., 2007; Sánchez et al., 2020), affect inhibitor binding (Tran et al., 2020), be present in Pol epitopes interacting with the immune system (Los Alamos National Laboratory, 2022a), or affect protein structure and conformation (Bandaranayake et al., 2008; Coman et al., 2008a,b; Kear et al., 2009). For example, some HIV-1 variants in our study (group O, subtype J, CRF13_cpx, 19_cpx, 49_cpx, and 51_01B, see Table 2) presented V-markers within the PR flaps (PR residues 37-71), the regions mediating accessibility of substrate to the PR active site (Hornak et al., 2006). However, the impact of these V-markers on the flap conformational changes of the corresponding variant is still unknown. Further research is required to evaluate the impact of the different levels of aa conservation in the PR, RT, and IN across HIV-1 variants and to evaluate the influence of each specific V-markers found at Pol in the viral replication cycle, protein structure, and function, as well as in the interactions with antiretroviral drugs or with the immune system.

## Data Availability Statement

The original contributions presented in this study are included in the article/Supplementary Material, further inquiries can be directed to the corresponding author. The datasets analyzed for this study can be found in the Los Alamos National Laboratory database (https://www.hiv.lanl.gov).

## Ethics Statement

The viral sequences were retrieved from public databases, and no human studies or animal studies were performed in this manuscript.

## Author Contributions

PT-H analyzed the HIV Pol LANL sequences, validated some EpiMolBio functions necessary for the sequences analyses, performed the computations, discussed results, and wrote the first version of the manuscript. RR downloaded and aligned the HIV Pol LANL sequences, developed the in-house EpiMolBio bioinformatics program, and validated the EpiMolBio functions necessary for the sequences analyses. AH designed and supervised the study, discussed results, reviewed and edited the manuscript, and applied for funding, being responsible for project administration. All authors approved the submitted final version.

## Conflict of Interest

The authors declare that the research was conducted in the absence of any commercial or financial relationships that could be construed as a potential conflict of interest.

## Publisher’s Note

All claims expressed in this article are solely those of the authors and do not necessarily represent those of their affiliated organizations, or those of the publisher, the editors and the reviewers. Any product that may be evaluated in this article, or claim that may be made by its manufacturer, is not guaranteed or endorsed by the publisher.

## Footnotes

1
https://hivdb.stanford.edu/hivdb

2
https://www.hiv.lanl.gov

3
https://unstats.un.org

4
https://www.mapchart.net

5
https://hivdb.stanford.edu/

6
https://hivdb.stanford.edu/hivdb/by-mutations/
